# Increased Expression of the Autocrine Motility Factor is Associated With Poor Prognosis in Patients With Clear Cell–Renal Cell Carcinoma

**DOI:** 10.1097/MD.0000000000002117

**Published:** 2015-11-20

**Authors:** Giuseppe Lucarelli, Monica Rutigliano, Francesca Sanguedolce, Vanessa Galleggiante, Andrea Giglio, Simona Cagiano, Pantaleo Bufo, Eugenio Maiorano, Domenico Ribatti, Elena Ranieri, Margherita Gigante, Loreto Gesualdo, Matteo Ferro, Ottavio de Cobelli, Carlo Buonerba, Giuseppe Di Lorenzo, Sabino De Placido, Silvano Palazzo, Carlo Bettocchi, Pasquale Ditonno, Michele Battaglia

**Affiliations:** From the Department of Emergency and Organ Transplantation—Urology, Andrology and Kidney Transplantation Unit, University of Bari, Bari (GL, MR, VG, AG, SP, CB, PD, MB); Department of Pathology, University of Foggia, Foggia (FS, SC, PB); Department of Pathology, University of Bari (EM); Department of Basic Medical Sciences, Neurosciences and Sensory Organs, University of Bari, Bari (DR); Department of Medical and Surgical Sciences, Clinical Pathology Unit, University of Foggia, Foggia (ER); Department of Emergency and Organ Transplantation—Nephrology, Dialysis and Transplantation Unit, University of Bari, Bari (MG, GL); Department of Urology, European Institute of Oncology, Milan (MF, OdC); and Department of Clinical Medicine, Medical Oncology Unit, Federico II University, Naples, Italy (CB, GDL, SDP).

## Abstract

Glucose-6-phosphate isomerase (GPI), also known as phosphoglucose isomerase, was initially identified as the second glycolytic enzyme that catalyzes the interconversion of glucose-6-phosphate to fructose-6-phosphate. Later studies demonstrated that GPI was the same as the autocrine motility factor (AMF), and that it mediates its biological effects through the interaction with its surface receptor (AMFR/gp78). In this study, we assessed the role of GPI/AMF as a prognostic factor for clear cell renal cell carcinoma (ccRCC) cancer-specific (CSS) and progression-free survival (PFS). In addition, we evaluated the expression and localization of GPI/AMF and AMFR, using tissue microarray-based immunohistochemistry (TMA-IHC), indirect immunofluorescence (IF), and confocal microscopy analysis.

Primary renal tumor and nonneoplastic tissues were collected from 180 patients who underwent nephrectomy for ccRCC. TMA-IHC and IF staining showed an increased signal for both GPI and AMFR in cancer cells, and their colocalization on plasma membrane. Kaplan–Meier curves showed significant differences in CSS and PFS among groups of patients with high versus low GPI expression. In particular, patients with high tissue levels of GPI had a 5-year survival rate of 58.8%, as compared to 92.1% for subjects with low levels (*P* < 0.0001). Similar findings were observed for PFS (56.8% vs 93.3% at 5 years). At multivariate analysis, GPI was an independent adverse prognostic factor for CSS (HR = 1.26; *P* = 0.001), and PFS (HR = 1.16; *P* = 0.01).

In conclusion, our data suggest that GPI could serve as a marker of ccRCC aggressiveness and a prognostic factor for CSS and PFS.

## INTRODUCTION

Renal cell carcinoma (RCC) accounts for approximately 2% to 3% of all adult malignancies, the highest incidence being in Western countries. This disease comprises different histopathological entities with specific clinical and biological characteristics. Clear cell RCC (ccRCC) is the most common subtype, accounting for 85% to 90% of renal malignancies. Recent estimates have calculated that in 2015, 61,560 new cases will be diagnosed (3.7% of all new cancer cases: 38,270 in men and 23,290 in women) and 14,080 patients (2.4% of all cancer deaths: 9070 men and 5010 women) will die of RCC in the United States.^1^ Not only do nearly 30% of patients with RCC present with metastatic disease, but also up to 30% of patients who undergo surgery with curative intent will relapse with disseminated disease.^2^ Recent studies have provided additional insight into the molecular and cellular mechanisms involved in RCC development and resistance to novel-targeted therapies.^3–5^ Considering the natural history of this disease, we need to identify novel biomarkers for early detection, risk assessment, prediction of clinical outcome, and treatment response. A prognostic role has been evaluated for several circulating biomarkers associated with different features of RCC biology, including carbonic anhydrase IX, C-reactive protein, CA15-3, and some cancer metabolism-related proteins.^6–11^

The introduction of high-throughput techniques has led to a more in-depth understanding of molecular bases underlying the development of urologic cancers, as well as the identification of novel biomarkers and potential therapeutic targets.^12–18^ In this setting, analysis of the cancer metabolome has shown that tumor cells require a reprogramming of the cellular energy metabolism in order to support continuous cell growth and proliferation.^19^

RCC is fundamentally a metabolic disease.^20^ Many studies have suggested that an altered metabolism is involved in the development of this tumor.^21–23^ In addition, it has been shown that many genes implicated in the RCC pathogenesis play an important role in controlling cell metabolism.^24,25^

**FIGURE 1:**
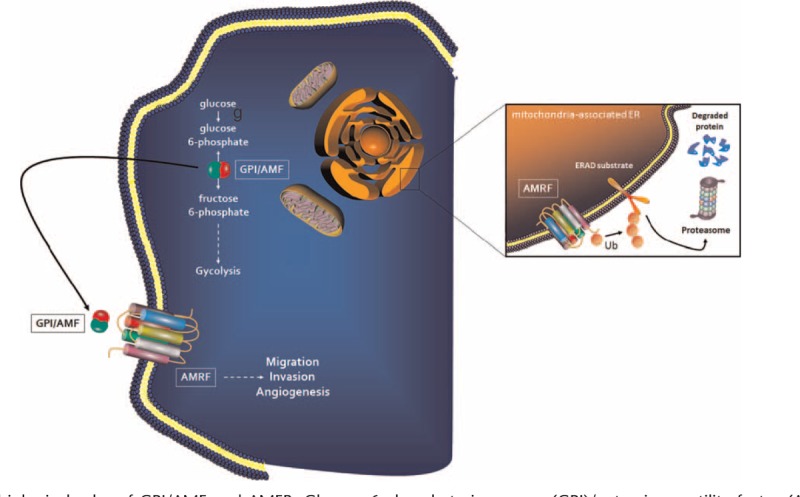
The biological roles of GPI/AMF and AMFR. Glucose-6-phosphate isomerase (GPI)/autocrine motility factor (AMF), is the second glycolytic enzyme that catalyses the interconversion of glucose-6-phosphate to fructose-6-phosphate. Moreover, GPI/AMF is involved in tumor cell migration, invasion, and angiogenesis, and these biological effects are mediated through the interaction with its surface receptor (AMFR/gp78). In the mitochondria-associated endoplasmic reticulum (ER), AMFR is also an E3 ubiquitin (Ub) ligase which is involved in the ER-associated protein degradation (ERAD) by cytosolic proteasomes.

Glucose-6-phosphate isomerase (GPI), also known as phosphoglucose isomerase, was initially identified as the second glycolytic enzyme that catalyzes the interconversion of glucose-6-phosphate to fructose-6-phosphate. Later studies demonstrated that G6PI and the autocrine motility factor (AMF) were the same, and that it mediated its biological effects through the interaction with its surface receptor (AMFR/gp78)^26^ (Fig. 1).

Recently, we showed that the flux of sugars through the pentose phosphate pathway (PPP), in association with the upregulation of some glucose metabolism-related enzymes including GPI, promoted both cancer cell proliferation and migration, as well as anabolic reactions in ccRCC.^9^ In the present study, we assessed the role of GPI/AMF as a prognostic factor for ccRCC cancer-specific (CSS) and progression-free survival (PFS). In addition, we evaluated the expression and localization of GPI/AMF and its surface receptor (AMFR/gp78), using tissue microarray-based immunohistochemistry (TMA-IHC), indirect immunofluorescence (IF), and confocal microscopy analysis.

## MATERIALS AND METHODS

### Study Population and Tissue Collection

Primary renal tumor (n = 180) and nonneoplastic tissues (n = 20) were collected from 180 patients who underwent radical or partial nephrectomy for ccRCC between January 2007 and December 2014. Two pathologists confirmed the presence of ccRCC in the neoplastic tissues and excluded tumor cells in the healthy specimens. Detailed clinical and pathological characteristics of the patients are summarized in Table 1. All patients were preoperatively staged by thoracoabdominal computed tomography or magnetic resonance imaging. Tumor staging was reassigned according to the seventh edition of the AJCC-UICC TNM classification. The 2004 World Health Organization and Fuhrman classifications were used to attribute histological type and nuclear grade, respectively. Written informed consent to take part was given by all participants. The protocol for the research project has been approved by the local Ethics Committee and conforms to the provisions of the Declaration of Helsinki in 1995.

**TABLE 1:**
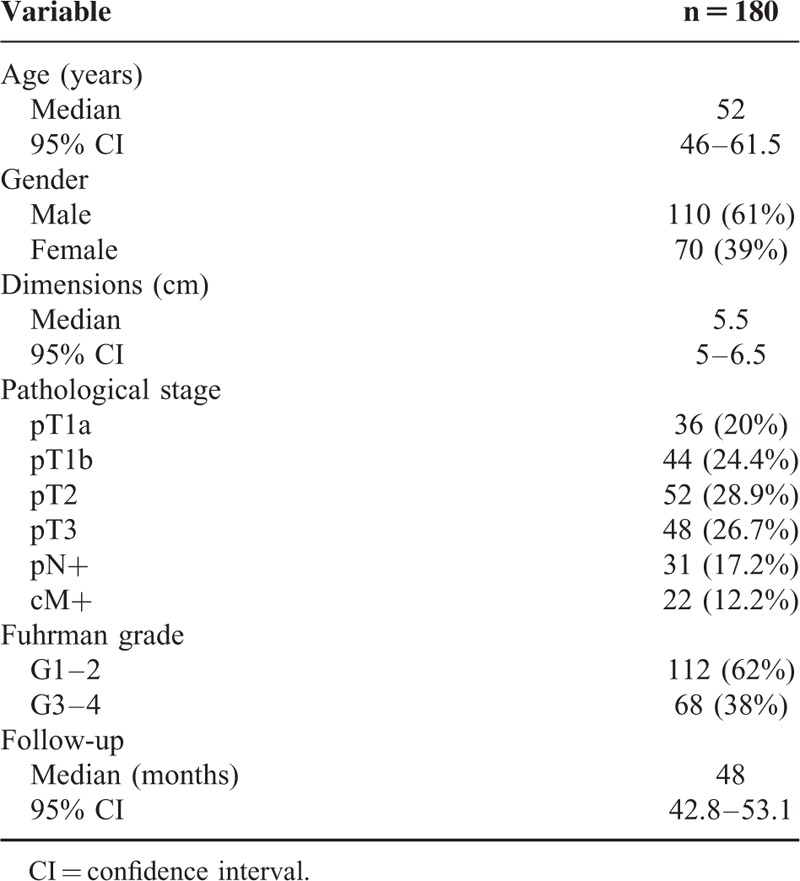
Clinical and Pathological Characteristics

### Real-Time PCR

Total RNA of normal and tumor tissue were reverse transcribed with the high-capacity cDNA reverse transcription kit (Applied Biosystems, Foster City, CA), following the manufacturer's instructions. Quantitative real-time polymerase chain reactions (PCR) were performed using iQTM SYBR Green Supermix buffer (6 mM MgCl_2_, dNTPs, iTaq DNA polymerase, SYBR Green I, fluorescein, and stabilizers) (BIO-RAD Laboratories, Hercules, CA). The following primers were used for real-time PCR: 5′-GATCCTCCTGGCCAACTTCT-3′ and 5′-GTTGGTTGGGCGATTTCCTT-3′ for GPI/AMF; 5′-AATCTGGCACCACACCTTCT-3′ and 5′-AGCCTGGATAGCAACGTACA-3′ for β-actin. Quantification of the mRNA levels was performed on a MiniOpticon real-time PCR detection system (BIO-RAD Laboratories). In the PCR reactions, the following protocol was used: activation of the polymerase at 95°C for 3 minutes, followed by 45 cycles at 95°C for 10 seconds, 60°C for 30 seconds. Melting curves were generated through 60 additional cycles (65°C for 5 seconds with an increment of 0.5°C/cycle). Gene expression results were obtained as mean Ct (threshold cycle) values of triplicate samples. Expression was determined using the 2^−ΔΔCt^ method. Expression values were normalized to β-actin.

### Data Mining Using Oncomine Gene Expression Microarray Datasets

GPI gene expression was analyzed using microarray gene expression datasets deposited in the Oncomine database (https://www.oncomine.org/resource/login.html). Firstly, to address the differential expression of GPI/AMF between renal cancer and normal tissues, combined filters were applied to display the corresponding datasets. The Cancer Type was defined as Clear Cell Renal Cell Carcinoma; Data Type was mRNA; Analysis Type was Cancer versus Normal Analysis.

The expression values of the GPI/AMF gene (log_2_ median-centered intensity) were read from the displayed bar chart. Student *t* test was used to calculate the significance.

### Quantification of G6PI/AMF Protein in Tissue Lysates

The MILLIPLEX MAP Human Glycolysis Pathway Magnetic Bead Panel (HGPMAG-27K, Millipore, Billerica, MA) was applied in 96-well plates for the quantification of GPI/AMF in tissue lysates. For the immunoassay procedures, 25 μl of each dilute lysate sample in assay buffer (5 μg total protein/well) and HeLa cells lysate (positive control) were added into wells in duplicate, according to the manufacturer's instructions. To each well, 25 μl of the mixed beads was added and the plate was incubated for 2 hours at room temperature. Human glycolysis pathway detection biotinylated antibodies were added for 1 hour; each captured a specific bead. After that, the reaction mixture was incubated for 30 minutes with streptavidin–PE conjugate to complete the reaction on the surface of each microsphere. Finally, the MILLIPLEX MAP was analyzed by Luminex xMAP technology. The immunoassay on the surface of each fluorescent-coded magnetic bead, MagPlex-C microsphere, was identified and quantified based on fluorescent signals. The median fluorescence intensity (MFI) was read with the Luminex 200 instrument and measured with xPONENT software.

### Immunohistochemistry and Tissue Microarray Construction

Nine high-density tissue microarrays (TMAs) were used for GPI/AMF and AMFR immunostaining. Archived formalin-fixed paraffin-embedded nephrectomy tissue samples for 180 cases were obtained. All tumor cores were identified by 2 uropathologists. These were selected by identifying representative tumor-containing slides and were used to assign the original tumor grade in each case. Three-millimeter cores were removed from the selected area (region of interest) using a needle punch. These 3-mm donor cores were subsequently embedded in previously arranged recipient paraffin blocks through a precisely spaced 15-hole array pattern. Core positions in the recipient paraffin block were noted on a TMA map. After paraffin cooling, the recipient blocks were cut in the microtome and used for immunohistochemistry. Immunohistochemical evaluation of GPI/AMF and AMFR protein expression was carried out on paraffin-embedded tissue sections. TMA were deparaffinized and rehydrated through xylene and graded alcohol series. Slides were subjected to specific epitope unmasking by microwave treatment (700 W) in citrate buffer (0.01 M pH 6.0). After antigen retrieval, TMA were incubated for 10 minutes with 3% H_2_O_2_ to block endogenous peroxidase activity. Sections were treated with serum-free protein block (Dako Cytomation, Glostrup, Denmark) at room temperature (RT) for 10 minutes and then incubated: at 4°C overnight with a mouse anti-AMF (1:200, Novus Biologicals, Littleton, CO) and at room temperature for 2 hours with a rabbit anti-AMFR (1:100, Novus Biologicals). Binding of the secondary biotinylated antibody was detected by the Dako Real EnVision Detection System, Peroxidase/DAB kit (Dako Cytomation), according to the manufacturer's instructions. Sections were counterstained with Mayer's hematoxylin (blue) and mounted with glycerol (Dako Cytomation). Negative controls were obtained by incubating serial sections with the blocking solution and then omitting the primary antibodies. Staining of histological sections was evaluated by optical light microscope using a Leica microscope fitted with a Coolpix 990 digital camera (Nikon). Protein immunoreactivity was scored on the extent and intensity of staining, which was graded on an arbitrary scale ranging from 0 to 3, with 0 = negative, 1 = low, 2 = medium, and 3 = high expression.

### Immunofluorescence and Confocal Laser Scanning Microscopy

Paraffin-embedded kidney sections were double-stained for GPI/AMF (1B7D7, Novus Biologicals) and AMFR (Novus Biologicals). The expression and localization of proteins was evaluated by indirect immunofluorescence and confocal microscopy analysis. After antigen unmasking, the sections were blocked with 2% BSA in PBS for 1 h at room temperature. Sections were incubated overnight at 4°C with a primary antibody against GPI (1:200 in blocking), followed by incubation for 2 hours with the secondary antibody Alexa Fluor 555 goat anti-mouse (1:200; Molecular Probes, Eugene, OR). Sections were washed in PBS and then incubated for 2 hours with primary antibodies against AMFR (1:100 in blocking) followed by incubation for 1 hour at 37°C with the secondary antibody goat anti-rabbit IgG FITC (Novus Biologicals). All sections were counterstained with TO-PRO-3 (Molecular Probes). Negative controls were performed by omitting the primary antibodies. Specific fluorescence was acquired by a Leica TCS SP2 (Leica, Wetzlar, Germany) confocal laser-scanning microscope using a ×63 objective lens.

### Statistical Analysis

Statistical calculations were performed with MedCalc 9.2.0.1 (MedCalc software, Mariakerke, Belgium) and PASW 18 software (PASW 18, SPSS, Chicago, IL). Comparisons of median protein values between different groups were evaluated by Mann–Whitney *U* test. Receiver operating characteristic (ROC) curve analysis was performed to identify the GPI protein expression cutoff for survival stratification.

In the CSS analysis, patients still alive or lost to follow-up were censored, as well as patients who died of RCC-unrelated causes. PFS was calculated from the date of surgery to the date of disease recurrence. Estimates of CSS and PFS were calculated according to the Kaplan–Meier method and compared with the log-rank test. Univariate and multivariate analyses were performed using the Cox proportional hazards regression model to identify the most significant variables for predicting CSS and PFS. A backward selection procedure was performed with removal criterion *P* > 0.10 based on likelihood ratio tests. Spearman test was applied to evaluate the correlations between GPI and tumor stage/size/grade. A *P*-value of <0.05 was considered statistically significant. Influence of the GPI on the predictive accuracy of the multivariate models was determined by Harrell concordance index.

## RESULTS

### GPI/AMF Expression Is Increased in Tumor Tissue and Is a Risk Factor for RCC Progression and Mortality

**FIGURE 2:**
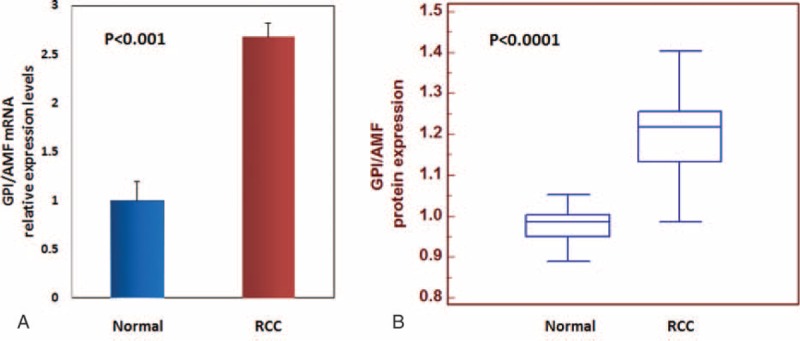
GPI/AMF gene expression (Panel A) and protein levels (Panel B) evaluated by real-time PCR and Luminex xMAP® technology, respectively. Normalized GPI/AMF mRNA and protein levels were significantly higher in clear cell renal cell carcinoma (RCC) as compared with normal tissue.

Detailed clinical and pathological characteristics of the patients are summarized in Table 1. To analyze the transcription levels, we firstly performed quantitative real-time PCR and evaluated GPI/AMF mRNA levels in ccRCC tissue samples, compared with normal renal parenchyma. GPI/AMF mRNA levels are shown in Figure 2A. Normalized gene expression levels for GPI/AMF were significantly higher in the ccRCC compared with the normal tissue. To confirm the above findings we analyzed the differential expression of GPI/AMF mRNA between renal cancer tissues and normal tissues by data mining of the Oncomine microarray gene expression datasets^27–31^ (Table 2). Overall, we found that GPI/AMF expression was significantly upregulated in ccRCC comparing with normal tissues.

**TABLE 2:**
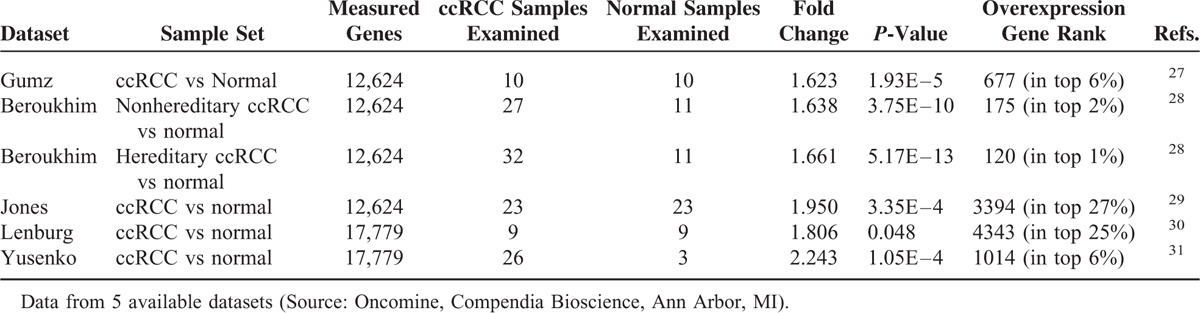
GPI/AMF Expression in ccRCC

**FIGURE 3:**
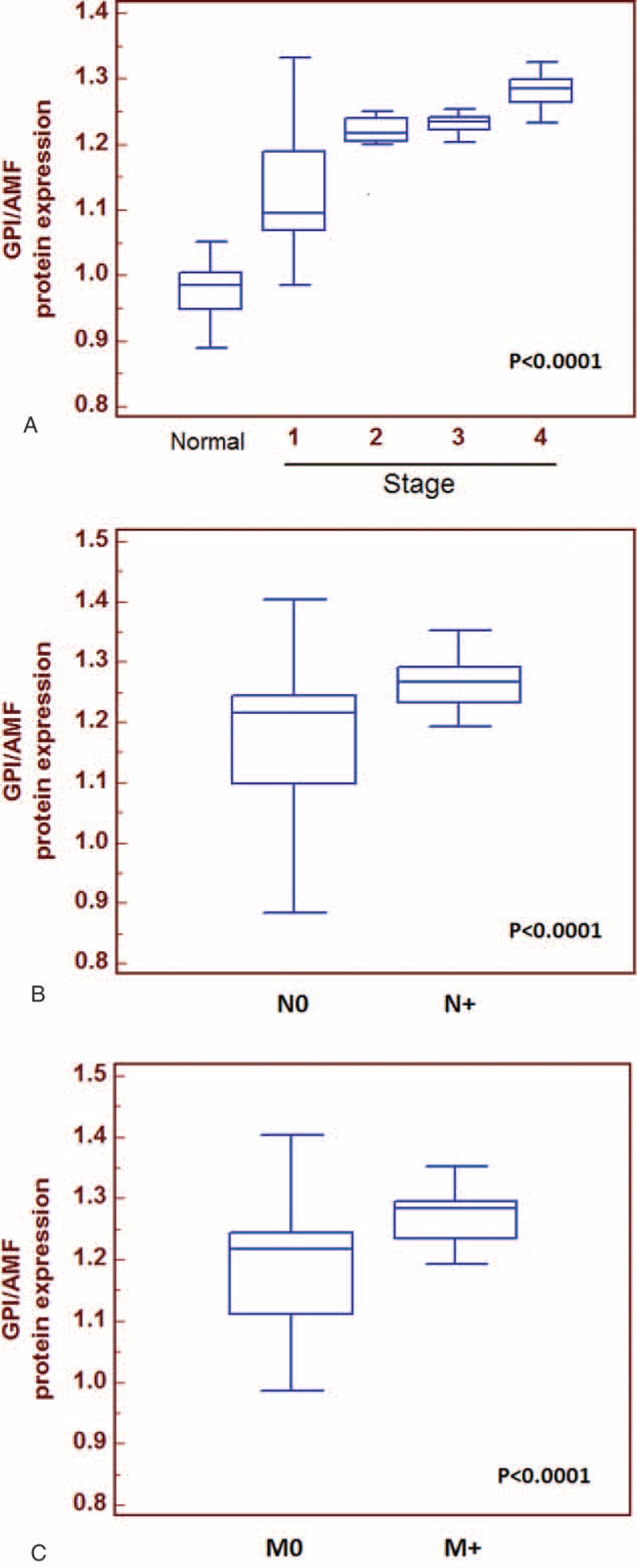
Comparisons of tissue GPI median values stratified according to clinical stage (Panel A) and between patients with or without lymph node metastases (Panel B) and with or without visceral metastases (Panel C). GPI/AMF median values were significantly higher in patients with advanced disease, with lymph node involvement and visceral metastases.

Next, we analyzed the GPI protein levels and the results were consistent with the gene expression levels. In particular, GPI protein levels were significantly higher in RCC patients than in healthy subjects (*P* < 0.0001) (Fig. 2B). Statistically significant differences resulted between GPI values and clinical stage (*P* < 0.0001; Spearman correlation: r_s_ = 0.61, *P* < 0.0001), lymph node involvement (*P* < 0.0001), and visceral metastases (*P* < 0.0001) (Fig. 3). No correlation was found between protein levels and tumor size (*P* = 0.38) or Fuhrman grade (*P* = 0.48).

**FIGURE 4:**
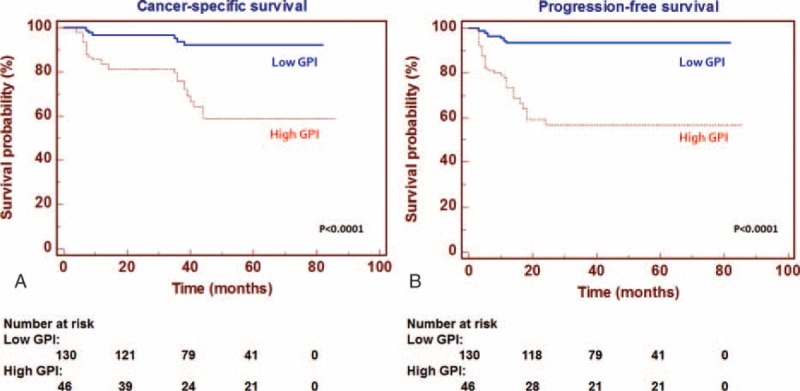
Kaplan–Meier cancer-specific survival (CSS) and progression-free survival (PFS) curves, stratified by GPI/AMF tissue levels. Patients with high tissue levels of GPI/AMF had reduced CSS (Panel A) and PFS (Panel B) as compared with patients with lower values.

To evaluate the association between patients survival and the expression levels of GPI (protein expression in tissue lysates), we classified the entire population by high versus low expression levels according to the cut-offs obtained with ROC curve analysis. After a median follow-up of 48 months (95% CI: 42.8–53.1), 20 patients had died of ccRCC. Kaplan–Meier survival curves for CSS and PFS, stratified by the GPI tissue levels, are shown in Figure 4. Both CSS and PFS were significantly decreased in patients with high levels of GPI/AMF. Univariate analysis for the predefined variables showed that pathological stage, presence of nodal and visceral metastases, Fuhrman grade, presence of necrosis, tumor size, and high levels of GPI were significantly associated with the risk of death (Table 3) and progression (Table 4). At multivariate analysis by Cox regression modeling, pathological stage, presence of nodal and visceral metastases, Fuhrman grade, and high levels of GPI, were independent adverse prognostic factors for CSS (Table 3) and PFS (Table 4). Regarding CSS, the c-index of the multivariate model without GPI was 0.81 compared with 0.84 when GPI was supplemented. For PFS, the c-index improved from 0.73 compared with 0.77 when the GPI was added.

**TABLE 3:**
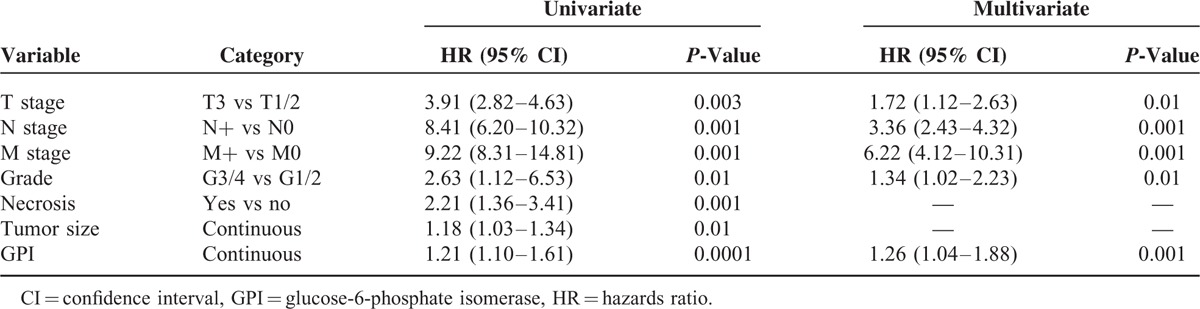
Univariate and Multivariate Analyses for Cancer-Specific Survival

**TABLE 4:**
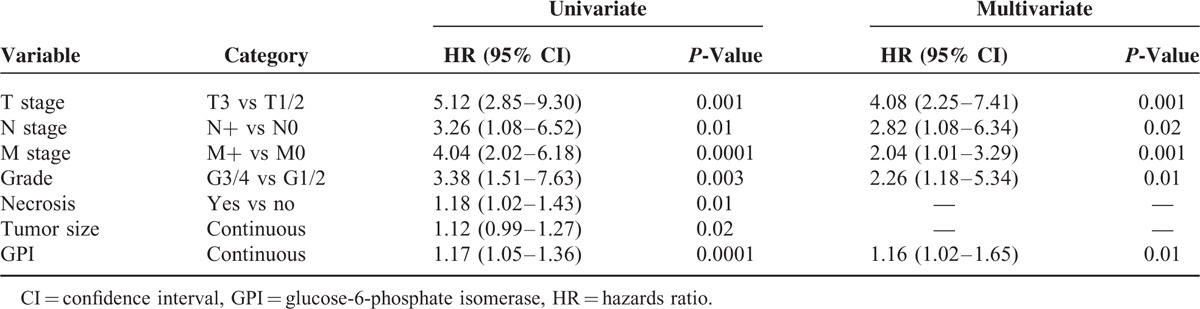
Univariate and Multivariate Analyses for Progression-Free Survival

### Distribution Pattern of GPI/AMF and AMFR in Normal and Tumor Tissues

**FIGURE 5:**
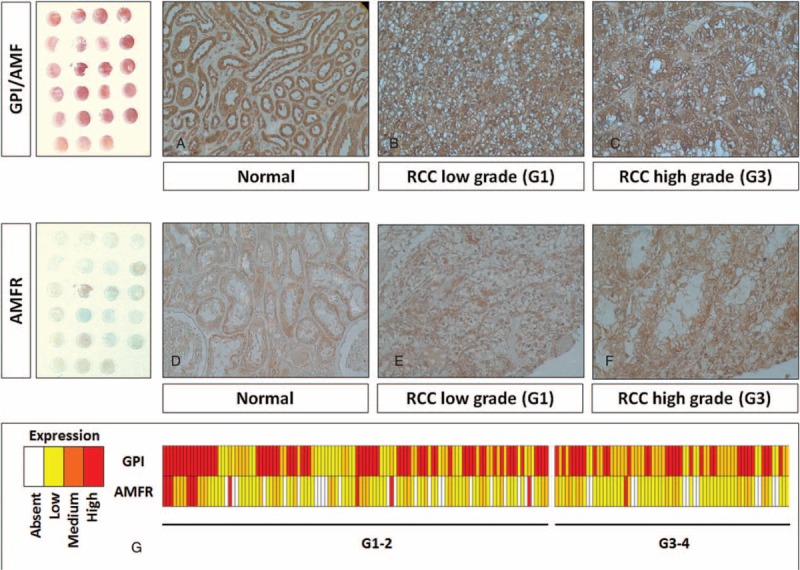
Immunohistochemical staining of GPI/AMF and AMFR proteins in tissue microarrays of human clear cell renal cell carcinoma (RCC) specimens. In normal kidney, GPI was predominantly localized in the cytoplasm of renal tubule cells, whereas it was absent in the glomeruli (Panel A). Clear cell RCC showed a stronger staining in cancer cells, with both a cytoplasmic and membranous pattern (Panels B and C). Similarly, AMFR expression was very low in normal kidney (Panel D), but showed higher levels in tumor tissue (Panels E and F). Heat map summarizing GPI and AMFR staining in 180 RCC patients (Panel G). Original magnifications 20×.

Finally, to visualize the location and expression of GPI/AMF and AMFR, we performed immunohistochemistry on normal and pathological tissues, using high-density TMAs. Normal kidney showed weak staining for GPI, predominantly localized in the cytoplasm of renal tubule cells, whereas it was not detected in the glomeruli (Fig. 5A). Instead, ccRCC showed a stronger staining in cancer cells, with both a cytoplasmic and membranous pattern (Fig. 5B and C). Similarly, AMFR expression was very low in normal kidney (Fig. 5D), but showed higher levels in tumor tissue (Fig. 5E and F). TMA evaluation showed GPI protein expression in 100% of cases, with high expression levels in 93 (51.6%) cases. AMFR was expressed at high levels only in 11 (6.1%) cases and unlike GPI, the receptor was not detectable in 35 (19.4%) patients (Fig. 5G).

**FIGURE 6:**
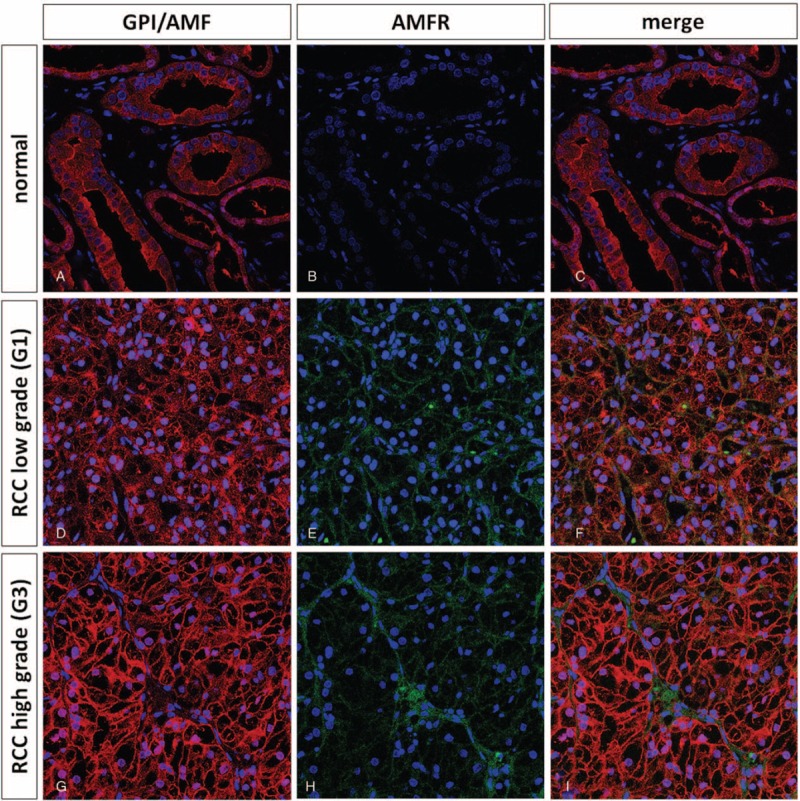
Immunofluorescence and confocal laser scanning microscopy of GPI/AMF and AMFR in normal (Panels A–C) and clear cell renal cell carcinoma (RCC) specimens (Panels D–I). Immunofluorescence staining showed an increased signal for both G6PI and AMFR in cancer cells, and their colocalization on plasma membranes (Panels F and I).

To confirm these findings, we analyzed GPI–AMFR coexpression in the normal and neoplastic renal tissue samples (Fig. 6). In particular, immunofluorescence staining showed an increased signal for both GPI and AMFR in cancer cells, and their colocalization on plasma membranes (Fig. 6D–I). Interestingly, GPI was detected both inside and outside the cancer cells, in accordance with its multifunctional role as a cytosolic enzyme and extracellular cytokine.

## DISCUSSION

GPI/AMF is a multifunctional protein that plays a dual role, both inside and outside the cell. Inside the cell, GPI functions as a cytosolic glycolytic enzyme that catalyzes the isomerization of glucose 6-phosphate to fructose 6-phosphate, and it is involved in the recycling of hexose-6-phosphate in the PPP. In addition to its role in cellular metabolism, outside the cell, GPI/AMF acts as a cytokine, and this function is dependent on the interaction with its membrane receptor, AMFR/gp78.^26,32^ In particular, GPI/AMF plays a role as a maturation factor for human myeloid leukemia cells, is a neurotrophic factor for embryonic spinal and sensory neurons, is involved in sperm agglutination, is a myofibril-bound serine proteinase inhibitor, and has a role in the development of somatosensory and motoric neural structures.^33–37^

AMFR/gp78 is a putative seven transmembrane G protein-coupled receptor that stimulates cell motility after binding with GPI/AMF. This protein is also located on the mitochondria-associated endoplasmic reticulum (ER) where it functions as a ubiquitin E3 ligase involved in the ER-associated degradation (ERAD) of proteins.

Many studies have shown that GPI/AMF and AMFR are overexpressed in some tumors, have a role in cancer progression and are negatively associated with patients’ clinical outcome.^9,38–40^ In a recent study, we explored the role of glycolysis and PPP in ccRCC and evaluated the activation of the GPI–AMFR axis in this tumor.^9^ In particular, we found that GPI was overexpressed in ccRCC, in association with high levels of glucose 6-phosphate and fructose 6-phosphate. Moreover, by in vitro and in vivo assays we showed that AMFR was involved in renal cancer cell migration, invasion, and tumor angiogenesis.^9^

In the present study performed in a large cohort of patients using quantitative real-time PCR and data mining of public Oncomine microarray datasets,^27–31^ we found that GPI/AMF mRNA was upregulated in ccRCC. Moreover, both the GPI/AMF and AMFR proteins were overexpressed in renal cancer tissue compared to normal kidney. In accordance with its biological role, GPI was also identified outside the tumor cells and costained with AMFR, indicating the colocalization with its membrane receptor.

Several reports have demonstrated that both GPI/AMF and AMFR expression are associated with a poor clinical outcome in some tumors. Jiang et al^38^ showed that GPI and AMFR were highly expressed in human breast cancer and were associated with reduced disease-free survival and CSS. Similarly, an increased GPI expression was associated with a higher metastatic potential in human lung carcinoma.^39^ In addition, a recent study showed that GPI/AMF levels were significantly increased in the serum and in neoplastic tissue of patients with endometrial carcinoma.^40^ In this scenario, to address the significance of GPI/AMF in ccRCC prognosis, we stratified the patients’ population according to protein expression levels. Kaplan–Meier curves showed significant differences in CSS and PFS between the patients groups with high versus low protein expression. In particular, patients with high tissue levels of GPI had a 5-year survival rate of 58.8%, as compared to 92.1% for subjects with low levels (*P* < 0.0001). Similar findings were observed for PFS (56.8% vs 93.3% at 5 years). These findings are in accordance with the results of other studies that showed how low expression of GPI contributes to the aggressive phenotype of different types of cancer cells.^41–43^

Multivariate analyses showed that high levels of GPI (HR = 1.26; *P* = 0.001) together with pT stage > 2, Fuhrman grade ≥3, and presence of nodal and visceral metastases, were significantly predictive of risk of death. Similarly, this marker remained an independent prognosticator of outcome in terms of PFS (HR = 1.16; *P* = 0.01). Some studies have demonstrated that GPI/AMF expression is associated with increased tumor cell motility and metastatic potential.^26,44,45^ In agreement with these results, we found increased levels of GPI in metastatic tumors compared to localized cancers. In fact, GPI protein expression levels were significantly increased in patients with lymph node (*P* < 0.0001) and visceral metastases (*P* < 0.0001). It has been shown that GPI–AMFR axis inhibition blocks the development of the metastatic phenotype and the migratory tumor cell capacity.^42^ In a recent study, we demonstrated that ccRCC cell treated with anti-AMFR antibody, had reduced migratory and invasive capabilities, and a decreased neoangiogenic response.^9^ Therefore, blocking this axis may serve as a putative therapeutic target for ccRCC.

The main limitations of this study include the single-center design of the report, and its retrospective nature.

In conclusion, the evidence supported the role of the GPI–AMFR axis in ccRCC progression, possibly via autocrine/paracrine mechanisms. Moreover, we provide a detailed description of GPI expression in ccRCC and discuss some clinical implications. In particular, we found that GPI could serve as a marker of ccRCC aggressiveness and a prognostic factor for CSS and PFS.
